# Robotic Lobectomy without Complete Fissure for Non-Small Cell Lung Cancer: Technical Aspects and Perioperative Outcomes of the Tunnel Technique

**DOI:** 10.3390/curroncol30060441

**Published:** 2023-06-19

**Authors:** Filippo Tommaso Gallina, Daniele Forcella, Enrico Melis, Francesco Facciolo

**Affiliations:** Thoracic Surgery Unit, IRCCS Regina Elena National Cancer Institute, 00144 Rome, Italy

**Keywords:** robotic-assisted thoracic surgery, NSCLC, lymphadenectomy, fissureless

## Abstract

Even though the use of the “fissure-last” technique in mini-invasive lobectomy with the fissureless condition is well accepted, in terms of perioperative outcomes, controversies still surround the hilar lymph node dissection. In this article, we reported a description of the robotic “tunnel technique” approach in the right upper lobectomy in the absence of a defined fissure. We then compared the short terms outcomes of 30 consecutive cases treated using this technique, with 30 patients treated using the “fissure last” VATS approach in the same institution, before the start of the robotic surgery program.

## 1. Introduction

The field of oncological thoracic surgery has witnessed a remarkable transformation with the introduction of robotic surgery [1,2]. This advanced technology offers several advantages, including high-definition visualization, increased flexibility of robotic arms, and tremor filtration. These features contribute to improved surgical precision, reduced trauma, and enhanced safety, particularly for complex thoracic procedures [3]. Furthermore, the utilization of robotic surgery not only aims to enhance surgical outcomes but also holds potential in achieving better oncological results, particularly in terms of achieving an optimal R status, which refers to the completeness of tumor resection [4,5].

In this study, we aim to provide a detailed description of the surgical technique for robotic right upper lobectomy, emphasizing the unique approach of initiating the dissection by first approaching the fissure, even in cases where a defined fissure plane is absent. This innovative “fissure-first” approach allows for meticulous exploration and dissection of the fissure vessel plane, enabling precise identification of anatomical structures and facilitating subsequent steps of the lobectomy procedure. By adopting this technique, we aim to optimize the surgical procedure and potentially improve patient outcomes.

To further evaluate the clinical benefits of the robotic “tunnel technique” approach, we conducted a case-matched comparison with the well-established, video-assisted thoracic surgery (VATS) technique. Despite few studies reporting the fissure-first approach using the thoracoscopic technique [6,7], the VATS approach traditionally employs a “fissure-last” technique, wherein the fissure is divided towards the end of the lobectomy procedure [8,9]. By comparing the outcomes between these two approaches, we aim to assess the potential advantages of the tunnel technique in terms of surgical efficacy, postoperative outcomes, and oncological parameters.

Through this report, we seek to contribute to the growing body of evidence on the application of robotic surgery in thoracic oncology and shed light on the potential of the “tunnel technique” approach as an innovative and promising strategy. By exploring and analyzing these advancements in robotic thoracic surgery, we hope to pave the way for further improvements in surgical techniques and ultimately enhance patient care and outcomes in the field of thoracic oncological surgery.

## 2. Materials and Methods

### 2.1. Study Design

The study was designed as a retrospective, single-center, case-matched analysis, comparing Video-Assisted Thoracic Surgery (VATS), thoracotomy, and Robotic-Assisted Thoracic Surgery (RATS) in patients with early stage non-small cell lung cancer (NSCLC), and clinical N0 disease, who underwent curative surgery. Data for the analysis were retrieved from our lobectomy database.

Until 2012, our standard surgical technique for treating early stage NSCLC was thoracotomy. In 2012, we initiated the VATS lobectomy program, initially employing the three-portal or bi-portal approach and transitioning to the uniportal technique in 2014. In 2016, we introduced the RATS lobectomy program using the Da Vinci robotic technology, starting with the Si model and subsequently adopting the Xi model.

The general inclusion criteria for this study comprised patients diagnosed with stage I-II NSCLC and clinical N0 disease, who underwent anatomical lobectomy with systematic lymph node dissection. Lymphadenectomy completeness was assessed in accordance with the IASLC definition, which mandates complete lymph node dissection of both N1 and N2 stations. Patients with a clinical stage III-IV disease, meant clinical N1 was confirmed via endoscopic procedures; small cell lung cancer (SCLC), sublobar resections, and wedge resections were excluded. Patients who received pre-operative chemotherapy or radiotherapy were also excluded.

In our study, we specifically compared the robotic fissure-first approach with the thoracoscopic fissure-last approach. This comparison was motivated by the standard practice observed in our institution, where robotic surgeons typically initiate the procedure by approaching the fissure first, while thoracoscopic surgeons tend to complete the fissure towards the end of the surgery. By investigating these distinct approaches, we aimed to gain insights into their respective outcomes and potentially identify any advantages or disadvantages associated with each technique.

From January 2020 to Dicember 2021, a total of 367 robotic procedures were performed at our institution, of which 240 were robotic lobectomy and radical lymphadenectomy for non-small cell lung cancer. After propensity matched score analysis, we selected 45 patients that underwent robotic lobectomy with the “fissureless” condition and we compared their results with 45 patients who underwent uniportal or biportal video-assisted thoracic surgery (VATS) lobectomy and radical lymphadenectomy performed at our institution.

### 2.2. Technique

**Figure 1:**
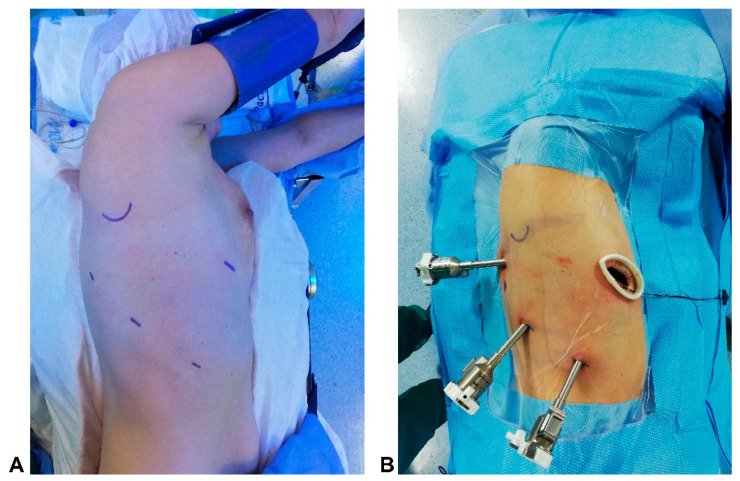
(**A**) Patient position; (**B**) Robotic ports placement;.

The operation was performed in the lateral decubitus and under general anesthesia using a double-lumen tube with single-lung ventilation (Figure 1A). The Da Vinci system is placed behind the patient. The four arms of the Da Vinci Xi Robot were connected to the thoracoports, performing three 8 mm incisions in the eighth intercostal space and an anterior utility incision of 3 cm in the fifth intercostal space (Figure 1B).

**Figure 2:**
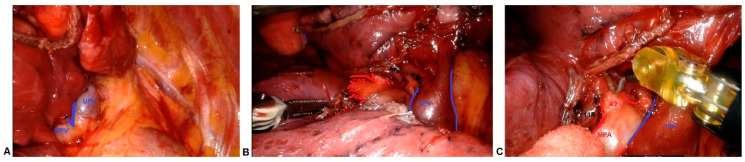
(**A**) upper pulmonary vein and middle pulmonary vein exposure; (**B**) pulmonary artery branches exposure; (**C**) hilar lymph nodes station 11RS.

The monopolar hook or the bipolar dissector was used by the right arm of the robot introduced through the incision utility. One of the forceps weas used to retract the lung and expose the structures. The other set of forceps were positioned on the left arm of the robot and used to grasp the structures during dissection. We started the dissection from the pulmonary ligament up to the inferior pulmonary vein. The posterior mediastinal pleura was opened until the upper lobe bronchus was fully exposed. Then, the anterior mediastinal pleura was opened and the pulmonary hilum was exposed. Therefore, the middle and the upper pulmonary veins were well identified (Figure 2A). The parenchyma of the middle lobe was divided from the upper lobe using an electronical stapler in the direction of the edge between the middle and upper pulmonary veins. Keeping close to the lower rim of the superior pulmonary vein, we continued to section the small fissure until the pulmonary artery plane was exposed. Once the middle pulmonary artery and the ascending artery were identified, sliding over this plane, the main fissure with the lower lobe was completed using an electronic stapler, revealing the lower segmental arterial branches (Figure 2B). After exposing the entire pulmonary artery plane, the hilar lymph nodes could be clearly identified and dissected, consequently performing an accurate hilar lymphadenectomy (Figure 2C).

At this point, all the arterial and venous vessels for the upper lobe were well exposed and could be divided using an electronic stapler. Lastly, the upper bronchus was divided, completing the lobectomy (Video S1, Supplementary Material).

### 2.3. Statistical Analysis

Statistical analyses were performed using the Statistical Package for Social Sciences for Windows (SPSS^®^, 23.0, Chicago, IL, USA). Non-parametric tests were used for comparisons and data were expressed as the median (standard deviation). Significance threshold was *p* < 0.05. To reduce the selection bias, the two populations were selected and matched one to one by a propensity score-matching analysis. For propensity score matching, the predicted probability of receiving RATS was calculated first by multivariable logistic regression, with the approach (RATS vs. VATS) as the binominal dependent variable, and age, sex, tumor site, body mass index (BMI), clinical TNM, as the independent factors. Next, patients in the RATS group were matched 1:1 with patients in the VATS group, having a difference in predicted probability equal or less to one quarter of the standard deviation of predicted probability.

## 3. Results

All the demographic and surgical features were reported in Table 1. Median age was 63 (44–77) years. Median hospital stay was 5 days (4–7) for the VATS group, while for the RATS group, 5 days (4–8) without significantly statistical differences. The postoperative major morbidity rate until 30 days after surgery was not significantly higher in the VATS group, compared to the robotic group (9, 20.0% vs. 7, 15.6%; *p* = 0.1). The postoperative air leaks occurred in four patients for the robotic group and three patients for the VATS group (*p* = 0.2). There was not 30 days mortality. The histopathological yield showed that the most frequent histological type was adenocarcinoma. The number of the hilar lymph nodes resected was significantly higher in the robotic than the VATS group (7, 4–15 vs. 4, 2–10; *p* = 0.04). We then analyzed the hilar nodal upstaging rate, the results showed that the tunnel technique group had a statistically significant and higher rate, compared to the VATS group (6, 13.3% vs. 2, 4.4%; *p* = 0.03). Moreover, by analyzing the single-hilar stations, a statistically significant higher number of station 12 lymph nodes are shown in the robotic group.

## 4. Discussion

The presence of a “fissureless” condition, particularly involving the horizontal fissure of the right lung, has been increasingly recognized in clinical practice. In traditional approaches to lung surgery, the exposure of the pulmonary artery involves opening the fissure and identifying segmental arterial branches corresponding to each lung lobe. However, in cases where the fissure is incomplete or absent, the intra-fissure identification of the pulmonary artery can pose challenges and increase the risk of postoperative, prolonged air leaks [10].

As the utilization of minimally invasive techniques, such as robotic surgery and VATS, has become more widespread in the treatment of non-small cell lung cancer (NSCLC), the “fissure-last” technique has gained popularity. This approach involves dividing the fissure using a mechanical or electronic stapler at the end of the lobectomy, aiming to prevent postoperative, prolonged air leaks [11]. However, a potential concern associated with the “fissure-last” technique is the limited exposure of the fissure vascular plane, which may result in a less accurate hilar lymphadenectomy. [12]

Multiple authors have highlighted the potential limitations of the “fissure-last” technique in achieving an accurate hilar lymphadenectomy, due to limited exposure of the fissure vascular plane. One study by Zhang et al. [13] emphasized that the fissure-first approach provides better visualization and facilitates complete dissection of hilar lymph nodes. Another study by Licht et al. [14] supported the advantages of the fissure-first technique in improving lymph node retrieval. Furthermore, Gossot et al. have advocated for the fissure-first approach, emphasizing its benefits in lymphadenectomy quality [15]. While the VATS fissure-last approach has gained popularity, authors promoting this technique also emphasize the importance of ensuring an accurate lymph node dissection, even if it requires opening an absent fissure.

Accurate lymph node dissection during lung surgery is crucial for achieving an optimal oncological outcome. Studies have demonstrated that patients with an uncertain R (resection) status, indicating incomplete lymph node dissection, have a worse prognosis compared to those with an R0 status. Therefore, ensuring a thorough lymph node dissection is essential to achieve an R0 status and improve patient outcomes [12].

Despite the importance of lymph node dissection in lung surgery, there is a paucity of studies in the literature that have specifically compared the “fissure-last” technique to the traditional technique in patients with a fissureless condition. Most available studies have analyzed small cohorts of patients without providing specific results regarding hilar lymph node dissection. Consequently, there remains a need to further investigate and compare the efficacy and safety of different surgical techniques in this particular patient population.

In our study, we aimed to address this gap in the literature by evaluating the outcomes of employing a tunnel technique approach with robotic technology during a robotic right upper lobectomy in patients with a fissureless condition. The rationale behind the “tunnel technique” approach is to allow for complete exposure of the fissure vessel plane before dissection, enabling a more accurate and thorough dissection of the hilar lymph nodes. This approach offers the potential advantage of removing a higher number of hilar lymph nodes while minimizing the risk of postoperative complications, including postoperative air leaks. Our short-term outcomes demonstrated promising results. By utilizing the “fissure-first” approach with robotic technology, we were able to achieve a higher lymph node yield without observing a corresponding increase in the incidence of postoperative complications. This finding suggests that the “fissure-first” approach may provide a safe and effective alternative for patients with a fissureless condition undergoing robotic right upper lobectomy.

The ability to remove a greater number of hilar lymph nodes is of significant clinical importance. Accurate staging of NSCLC is critical for appropriate treatment planning and determining patient prognosis. Furthermore, studies have shown that patients with a higher lymph node yield tend to have improved long-term survival outcomes compared to those with a lower lymph node yield. Therefore, the ability to achieve an accurate and thorough lymphadenectomy is of paramount importance in lung surgery [16,17].

In addition to the advantages of increased lymph node yield, the “tunnel technique” approach with robotic technology offers the advantage of potentially reducing the incidence of postoperative air leaks. Prolonged air leaks following lung surgery can lead to various complications, such as increased length of hospital stay, increased risk of infection, and delayed recovery. By utilizing electronic staplers to divide the fissure, the “tunnel technique” approach aims to minimize the risk of postoperative air leaks and optimize postoperative outcomes.

The absence of the fissure in lung cancer surgery can present challenges, particularly in terms of accurately staging the disease. The upstaging rate, which refers to the reclassification of a tumor to a higher stage based on additional pathological findings, is an important factor to consider in this context. When the fissure is absent, the traditional approach of relying on fissure dissection to identify segmental arterial branches and assess lymph node involvement becomes more challenging. This may lead to a higher upstaging rate, as the complete evaluation of lymph node status and the extent of disease becomes more complex. However, it is crucial to interpret the implications of the upstaging rate in the absence of the fissure with caution [18]. While upstaging may initially be perceived as a negative outcome, it provides an opportunity for a more comprehensive assessment of the disease. The identification of additional lymph node involvement or microscopic tumor spread can guide treatment decisions and potentially lead to more targeted and effective therapies [19]. Therefore, the higher hilar-upstaging rate in the absence of the fissure should be viewed as an opportunity for more tailored and aggressive treatment strategies, rather than a purely negative outcome. The distinction made in our study between the hilar lymphadenectomy and mediastinal lymphadenectomy is an important aspect to consider in the discussion. While our current paper focused on N1 upstaging and the impact of surgical approach in the hilar lymphadenectomy, it is crucial to acknowledge that the quality of mediastinal lymphadenectomy can also be influenced by the surgical technique, as demonstrated in our previous publication. Further investigations exploring the relationship between surgical approach and mediastinal lymphadenectomy quality are warranted to comprehensively evaluate the overall impact on nodal staging accuracy.

It Is important to continue exploring innovative approaches and techniques that can aid in accurate staging and ensure optimal patient outcomes in the absence of the fissure. Advances in imaging technologies, such as preoperative imaging modalities and intraoperative imaging guidance, may play a crucial role in improving the accuracy of staging and reducing the upstaging rate. Further research and larger-scale studies are needed to better understand the impact of upstaging in the absence of the fissure on long-term outcomes and survival rates. By gaining more insights into the factors contributing to upstaging, and exploring strategies to mitigate its impact, we can optimize the management and treatment of lung cancer patients, ultimately improving their prognosis and quality of life. While our study provides valuable insights into the benefits of the “fissure-first” approach in patients with a fissureless condition, there are several limitations to acknowledge. Firstly, our study had a relatively small sample size, and a larger-scale investigation involving multiple centers would be beneficial to validate our findings. Additionally, the long-term outcomes and survival data are lacking in our study, and further research with extended follow-up periods is necessary to evaluate the impact of the “tunnel technique” approach on overall patient outcomes.

The absence of the fissure in lung surgery presents challenges in terms of accurate staging and optimal treatment planning. The “fissure-last” technique has gained popularity to prevent postoperative air leaks, but it may compromise the accuracy of hilar lymphadenectomy. Our study focused on a “fissure-first” approach using robotic technology in patients with a fissureless condition. We demonstrated that this technique allows for a higher lymph node yield without increasing complications. Accurate lymphadenectomy is crucial for optimal outcomes in lung cancer patients. Further research is needed to explore innovative techniques and imaging modalities to enhance staging accuracy and improve long-term outcomes in this patient population.

## 5. Conclusions

In conclusion, our study suggests that the “tunnel technique” approach with robotic technology during a robotic right upper lobectomy in patients with a fissureless condition is a safe and effective technique. It allows for complete exposure of the fissure vessel plane before dissection and facilitates an accurate and thorough hilar lymphadenectomy. By utilizing electronic staplers to divide the fissure, the “fissure-first” approach aims to minimize the risk of postoperative air leaks while respecting the anatomical and lymphatic constraints. Future research with larger sample sizes and long-term follow-up is warranted to further investigate the clinical benefits of this approach and its impact on patient outcomes in the context of a fissureless condition.

## Figures and Tables

**Table 1:** Demographic features of 45 consecutive patients underwent robotic lobectomy using the “fissure-first” technique matched with 45 “fissure-last” VATS lobectomy.

Variables	RATS	VATS	*p*-Value
Age (years)			0.3
Median (range)	63.50 (47–79)	67.30 (49–78)	
Smoking history			0.5
Yes (%)	22 (73.3)	24 (80)	
No (%)	8 (26.7)	6 (20)	
Comorbidities			0.2
COPD (%)	14	12	
Cardiac diseases (%)	8	5	
Diabetes mellitus (%)	3	2	
Other cancers (%)	1	0	
Other disease (%)	5	7	
No (%)	19	9	
Duration of Surgery (min.)			0.1
Median (range)	147 (109–174)	131 (113–189)	
Daily drainage (mL)			0.09
Median (range)	182 (142–239)	171 (134–234)	
Median hospital stay (days)			0.6
Nr (range)	5 (4–8)	5 (4–7)	
Postoperative morbidity rate			0.5
Nr (%)	5 (16.7)	6 (20)	
Postoperative complication			
Air leaks (%)	4 (8.8)	3 (6.7)	
Pneumonia (%)	-	1 (2.2)	
Arrythmia (%)	3 (6.6)	2 (4.4)	
Others (%)	-	2 (4.4)	
Histology			0.6
Adenocarcinoma (%)	40 (88.8)	39 (86.6)	
Squamous (%)	6 (13.3)	5 (11.1)	
pT			0.5
T1 (%)	27 (60.0)	26 (57.7)	
T2 (%)	15 (33.3)	14 (31.1)	
T3 (%)	3 (6.6)	5 (11.1)	
pN			0.2
N0	36 (80.0)	39 (86.7)	
N1	8 (17.8)	6 (13.3)	
N2	1 (3.3)	0 (0)	
Hilar Lymph nodes resected			0.04
Median (range)	7 (4–15)	4 (2–10)	
Station 10	3 (2–4)	3 (2–4)	0.2
Station 11	5 (2–6)	3 (1–4)	0.07
Station 12	4 (1–6)	1 (1–2)	0.03
Hilar Upstaging rate			
Nr (%)	6 (13.3)	2 (4.4)	0.03

## Data Availability

Available on request.

## Supplementary Materials

The following supporting information can be downloaded at: https://www.mdpi.com/article/10.3390/curroncol30060441/s1, Video S1: Video S1.
